# Novel role of *Dipterocarpus tuberculatus* as a stimulator of focal cell adhesion through the regulation of MLC2/FAK/Akt signaling pathway

**DOI:** 10.1080/19336918.2022.2073002

**Published:** 2022-05-26

**Authors:** Su Jin Lee, Ji Eun Kim, Jae Won Jung, Yun Ju Choi, Jeong Eun Gong, Bounleuane Douangdeuane, Onevilay Souliya, Young Whan Choi, Sung Baek Seo, Dae Youn Hwang

**Affiliations:** a Department of Biomaterials Science (BK21 FOUR Program), College of Natural Resources and Life Science/Life and Industry Convergence Research Institute/Laboratory Animals Resources Center, Pusan National University, Miryang, Republic of Korea; b Department of products development, Institute of Traditional Medicine, Ministry of Health, Vientiane, Lao PDR; c Department of Horticultural Bioscience, Pusan National University, Miryang, Republic of Korea

**Keywords:** *Dipterocarpus tuberculatus*, focal cell adhesion, integrin, FAK, PI3K

## Abstract

To investigate a novel function of *Dipterocarpus tuberculatus* on focal cell adhesion stimulation, alterations to the regulation of focal cell adhesion-related factors were analyzed in NHDF cells and a calvarial defect rat model after treatment with methanol extracts of *D. tuberculatus* (MED). MED contained gallic acid, caffeic acid, ellagic acid, and naringenin in high concentrations. The proliferation activity, focal cell adhesion ability, adhesion receptors-mediated signaling pathway in NHDF cells were increased by MED. Also, a dense adhered tissue layer and adherent cells on MED-coated titanium plate (MEDTiP) surfaces were detected during regeneration of calvarial bone. The results of the present study provide novel evidence that MED may stimulate focal cell adhesion in NHDF cells and a calvarial defect rat model.

## Introduction

Enhancement of focal cell adhesion via the promotion of tissue integration around an implant is considered an essential factor for successful implantation [1]. During this process, cell adhesion to biomaterials can be affected by the chemistry and topography of the material’s surface, and various cellular responses, including cell proliferation, cell migration, cell morphology, cell survival, and gene expression, are altered by the interaction between cells and implant materials [2–7]. Also, proliferation and differentiation of adherent cells within the implanted tissue are required for extracellular matrix (ECM) anchorage [8]. To achieve these effects, various chemical and topological modifications have been applied to modern implants [9–11]. Among these modifications, osteoconductive cement, topographical modification, and immobilization of bioactive molecules have been applied to implant surfaces in order to reduce giant cell recruitment, osteolysis, and fibrous capsule formation [9,12,13]. In these studies, the applicability of polyphenol-immobilized coating has been investigated in various medical devices, including bone implants, cardiovascular stents, and drug delivery systems [14–16]. Actually, cell adhesion activities have been considered a key factor for the successful implantation of devices and biomaterials [17,18]. However, the regulation of focal adhesion formation has been considered a complex process that includes the turnover of single contacts and the reinforcement of the adhesion plaque [19]. This type of adhesion is observed in diverse protein networks, including the structural interaction between ECM and actin cytoskeleton, and sequentially leads to cell migration and spreading [19]. Furthermore, adhesion clusters can be linked to the activation of a signaling pathway that mediates cell survival, growth, and differentiation [19].

It has been reported that some natural products can regulate focal cell adhesion, and their inhibitory and stimulatory functions have been analyzed in different cell types. Mulberry water extracts (MWEs) inhibit the migration of vascular smooth muscle cells (VSMCs) through suppression of the integrin-β3/focal adhesion kinase complex [20]. Integrin-mediated focal adhesion and formation of F-actin stress in melanoma A375 cells were inhibited by treatment with a *Bupleurum chinense* polysaccharide [21]. Tea polyphenols inhibit the smooth muscle cell (SMC)–extracellular matrix (ECM) interaction through the regulation of the integrin β1 receptor and ECM proteins [22]. Also, enhancement of cell migration, induced by increases of p-FAK and p-ERK1/2, was observed in human umbilical vein endothelial cells after treatment with *Citrus unshiu* peel [23]. *Panax notoginseng* saponins induce a decrease in the integrin family’s expression and inhibit FAK phosphorylation [24]. However, most of those studies have been focused on the inhibition of focal cell adhesion as part of a potential therapy for tumor metastasis. Few studies have investigated the functions and action mechanism of natural products that stimulate cell-biomaterial interactions.

The current study was undertaken to describe a novel function of a methanol extract of *D. tuberculatus* leaves (MED) on the stimulation of focal cell adhesion. Specifically, the effect of MED was assessed in MED-treated MatTek’s Normal Human Dermal Fibroblasts (NHDF) and in a MED-coated titanium plate (MEDTiP)-implanted calvarial defect rat model.

## Materials and methods

### Preparation and extraction of MED

A lyophilized sample of MED (FBM 213–075) was obtained from the International Biological Material Research Center of the Korea Research Institutes of Bioscience and Biotechnology (Daejeon, Korea). Briefly, dried stem powder of *D. tuberculatus* Roxb. was mixed with methanol in a fixed liquor ratio (1:10, powder: water). The mixture was repetitively subjected to the following steps: sonication for 15 min, followed by incubation for 2 h ten times per day for 3 days, then filtered through a 0.4 µm pore size filter. Subsequently, the filtered methanol extract was concentrated using a Rotary Evaporator (N = 1000SWD, EYELA, Bohemia, NY, USA), and lyophilized using a Speed Vacuum Concentrator (Modulspin 40, Biotron Co., Marysville, WA, USA). The final MED sample was dissolved in dimethyl sulfoxide (DMSO, Duchefa Biochemie, Haarlem, Netherlands) to obtain concentrations appropriate for the experiments.

### High-performance liquid chromatography (HPLC) analysis and quantification

MED (31 mg) was dissolved with 1 mL MeOH and then filtered with a 0.45 μm syringe filter (Millipore, Darmstadt, Hesse, Germany). The sample was subjected to HPLC for quantitative analysis of gallic acid, cafffeic acid, ellagic acid, and naringenin. The HPLC analysis was carried out using an Agilent Technologies series 1100 Infinity HPLC system (Santa Clara, CA, USA) consisting of an auto-sampler, binary pump, column oven, and diode array detector. Sample separation was achieved using a 5 μm Luna C18(2) 100 Å column (250 × 4.6 mm) (Phenomenex, Torrance, CA, USA). The injection volume was 10 μL, and the measured bioactive compounds were detected at 254 nm. The mobile phase, which was composed of 0.025% formic acid in distilled water (solvent A) and 100% acetonitrile (solvent B), was applied at 0.5 mL/min at a column oven temperature of 30°C. Stock solutions of standards were prepared in 100% MeOH (Thermo Fisher Scientific Inc., Wilmington, DE, USA) or water. The compounds in the samples were identified by comparing their retention times with those of the standards and quantified using their calibration curves in the linear range.

### Cell viabilities

NHDF cells established from the dermis of juvenile foreskin or adult skin from different locations were purchased from the ATCC (Manassas, VA, USA). Cells were grown in a humidified 5% CO_2_ and 95% atmosphere at 37°C in Dulbecco’s modified Eagle’s medium (DMEM; Welgene, Gyeongsan-si, Korea) containing 10% fetal bovine serum (FBS), 2 mM glutamine, 100 U/mL of penicillin, and 100 μg/mL of streptomycin.

The viabilities of NHDF cells were determined using an MTT (3-[4,5-dimethylthiazol-2-yl]-2,5 diphenyltetrazolium bromide) assay (Sigma-Aldrich Co., St. Louis, MO, USA). Briefly, NHDF cells were seeded at a density of 3 × 10^4^ cells in 200 μL of DMEM and cultured for 24–48 h in a 37°C incubator. When cells had attained 70–80% confluence, they were treated with MED (100, 200 and 400 μg/mL). NHDF cells were divided into three groups; a vehicle (DMSO) group, a low MED concentration group (100 μg/mL; LMED group), a middle MED concentration group (200 μg/mL; MMED group), and a high MED concentration group (400 μg/mL; HMED group). After incubation for 24 h, culture supernatants were discarded, 200 μL of fresh DMEM and 50 μL of MTT solution (2 mg/mL in 1× PBS) were added per well, and the cells incubated at 37°C for 4 h. The formazan precipitates that formed were then dissolved in DMSO (Duchefa Biochemie). Absorbance of each well was assessed at 570 nm using a Versa max plate reader (Molecular Devices, Sunnyvale, CA, USA).

### Trypsin-based cell detachment assay

For the trypsin-based detachment assay, NHDF cells were seeded at a density of 3 × 10^4^ cells in 200 μL of DMEM and cultured for 24–48 h in a 37°C incubator. When cells had attained 70–80% confluence, they were treated with MED (100, 200 or 400 μg/mL) for 24 h. After incubation for 24 h, cells were incubated with trypsin/EDTA (0.25%) for 5 min, and the detached cells were discarded with 1×PBS washing. The average number of attached cells was determined in three different areas within each well.

### Cell migration assay

The wound-healing assay was conducted using a method previously described [25]. Briefly, NHDF cells were evenly seeded in 6-well plates and subsequently grown to 70–80% confluence. Following removal of the culture medium, a cell wound was artificially created using a sterile pipette tip. The detached cells and the associated debris were washed twice with 1× PBS buffer, and the attached cells were further incubated for 16 or 24 h in DMEM containing 100, 200 or 400 μg/mL of MED. The detached cells in the scratch area were observed at two different times (16 or 24 h) using a microscope (Leica Microsystems, Wetzlar, Germany) at 400× magnification. The wound closure rate was calculated using the following formula: Wound closure rate (%) = (Original width − Width after migration)/Original width × 100

### Scanning Electron Microscope (SEM) analyses

NHDF cells were evenly seeded in 8-well glass slide chambers (Lab-Tek®, Australia) and subsequently grown to 70–80% confluence. The cells were then treated with MED (100, 200 or 400 μg/mL) for 24 h. After discarding the supernatants, cells were fixed with 2.5% glutaraldehyde in 0.1 M phosphate buffer (pH 7.4) at 4°C overnight. The following day, the glutaraldehyde solution was removed, and the spheres were washed with 1× PBS. The spheres were frozen in a freezer overnight, then freeze‐dried overnight. Platinum was sputtered on the material to form an 8 nm thick layer on the surface. The SEM images were captured at 5.0 kV accelerating voltage using an HRLV-SEM (JEOL, Tokyo, Japan). The MEDTiP harvested from the calvarial defect model rats was treated with the same method used to prepare NHDF cell samples.

### F-actin staining

NHDF cells were evenly seeded in 6-well plates and subsequently grown to 70–80% confluence. The cells were then treated with MED (100, 200 or 400 μg/mL) for 24 h. After discarding the supernatants, cells were fixed for 20 min with 4% formaldehyde at room temperature. After fixation, the cells were washed with 1× PBS and permeabilized using 0.1% Triton-X in PBS. Then, cells were stained with F-actin staining Actin Red 555 Ready Probes (Thermo Fisher Scientific Inc.) in PBS and imaged using a fluorescent microscope (Evos m5000, Thermo Fisher Scientific Inc.) at 200× and 400× magnification.

### Western blot analysis

Total proteins were obtained from NHDF cells as well as skin, skull, and adherent tissues of the rat models by applying Pro-Prep Protein Extraction Solution (Intron Biotechnology Inc., Seongnam, Korea) in accordance with the manufacturer’s protocol. After centrifugation at 13,000 rpm/min for 5 min, protein concentrations were determined using a SMART™ Bicinchoninic Acid Protein Assay Kit (Thermo Fisher Scientific Inc). Proteins were separated by 4–20% sodium dodecyl sulfate-polyacrylamide gel electrophoresis(SDS-PAGE) for 2 h, and then transferred to nitrocellulose membranes at 40 V for 2 h. Membranes were then incubated at 4°C with the following primary antibodies overnight: anti-FAK (Cell Signaling Technology, Danvers, MA, USA), anti-p-FAK (Cell Signaling Technology), anti-MLC (Abcam, Cambridge, UK), anti-p-MLC (Abcam), anti-Fibronectin (Invitrogen, Waltham, Massachusetts, USA), anti-VEGF (Pepro Tech, New Jersey, US), anti-PI3K (Cell Signaling Technology), anti-p-PI3K (Cell Signaling Technology), anti-AKT (Cell Signaling Technology), anti-p-AKT (Cell Signaling Technology), anti- GSK 3β (Cell Signaling Technology), anti-p(S9)-GSK3β (Cell Signaling Technology), or anti-β-actin antibody (Cell signaling Technology). The membranes were then washed with washing buffer (137 mM NaCl, 2.7 mM KCl, 10 mM Na_2_HPO_4_, and 0.05% Tween 20) and incubated with 1:2,000 diluted horseradish peroxidase (HRP)-conjugated goat anti-rabbit IgG (Invitrogen) at room temperature for 1 h. Blots were developed using Amersham ECL Select Western Blotting detection reagent (GE Healthcare, Little Chalfont, UK). Chemiluminescence signals from specific bands were detected using FluorChemi®FC2 (Alpha Innotech Co., San Leandro, CA, USA).

### Quantitative real time-PCR (RT-qPCR) analysis

Total RNAs were purified from NHDF cells as well as from skin, skull, and adherent tissues of the rat model using RNAzol (Tet-Test Inc., Friendswood, TX, USA). After determining the total RNA concentrations, complementary DNA (cDNA) was synthesized using Invitrogen Superscript II reverse transcriptase (Thermo Fisher Scientific Inc.). The quantitative PCR was performed using the cDNA template (1 μL) and 2× Power SYBR Green (6 μL; Toyobo Life Science, Osaka, Japan) containing specific primers. The primer sequences used for target gene expression identification were as follows: E-cadherin, sense 5’- CAA AGT GGG CAC AGA TGG TGT G-3’ and anti-sense, 5’- CTG CTT GGA TTC CAG AAA CGG-3’, integrin αV, sense 5’- CTC TTC TGC CTG CAC TTT G-3’ and anti-sense, 5’- ATG GGC TAC AGG CTT GTC ACT C-3’, integrin α5, sense 5’-GTG GTG CTG TCT ACC TCT GTC CTT G-3’ and anti-sense, 5’-CAG TTC CAT CTA TGA CAG CTA CCT AG-3’, integrin β1, sense 5’- AAC GAG GTC ATG GTT CAT GTT G-3’ and anti-sense, 5’- CAA CGC CAA TAA GAA CAA TTC CA-3’, integrin β3, sense 5’- GTC CTC CAG CTC ATT GTT GAT G-3’ and anti-sense, 5’- GGT CAC GCA CTT CCA GCT CTA-3’. The qPCR was performed over 40 cycles of denaturation at 95°C for 15s, annealing at 70°C for 60s, and extension at 70°C for 60s. Fluorescence intensities were measured at the end of the extension phase of each cycle. Threshold values for sample fluorescence intensities were set manually, and reaction cycles in which PCR products exceeded the fluorescence intensity threshold during the exponential phase were considered as threshold cycles (Ct). Expressions of E-cadherin, integrin αV, integrin α5, integrin β1, and integrin β3 genes were quantified with respect to β-actin (the housekeeping gene) by comparing Ct values at a constant fluorescence intensity, as described by Livak and Schmittgen.

### Experimental design for animal study

The Pusan National University–Institutional Animal Care and Use Committee reviewed and approved the animal protocol used in this study (Approval No. PNU-2020-2743). The SD rats were housed at the Pusan National University-Laboratory Animal Resources Center, which is accredited by the Korean Food and Drug Administration (Unit 000231) and the Association for Assessment and Accreditation of Laboratory Animal Care International (unit 001525). Male SD rats (eight weeks old) were purchased from Samtako BioKorea Co. (Osan, Korea). Water and a standard irradiated chow diet (Samtako BioKorea Co.) were provided *ad libitum* to all animals throughout the experimental period. Furthermore, all rats were maintained under specific pathogen-free conditions (50 ± 10% relative humidity, 23 ± 2°C) under a strict light: dark cycle (lights on at 08:00 h and off at 20:00 h).

Briefly, 8-week-old SD rats (male, n = 10) were assigned to a non-treated Ti plate- implanted group (TiP-implanted group, n = 5) or an MED-coated Ti plate-implanted group (MEDTiP group, n = 5). TiP were cut into small squares (0.3 cm × 0.3 cm) and implanted in the skull. Prior to implanting, all TiP were washed with ethanol, and the debris was blown away with nitrogen gas. TiP was treated by applying ozone plasma for 3 min and immersion in a MED solution dissolved in DMSO at a concentration of 40 mg/mL overnight. After washing with DMSO, residual MED was blown away with nitrogen gas. Cranial defects on the bilateral sides of the midline in each animal were generated using a Dental micro drill (Strong204, SAESHIN, Daegu, Korea) with a diameter of 2 mm. These defects were then covered with TiP or MEDTiP (1 cm × 1 cm) that had been sterilized in 70% EtOH solution for 15 min (Figure 8(a)).

**Figure 1.**
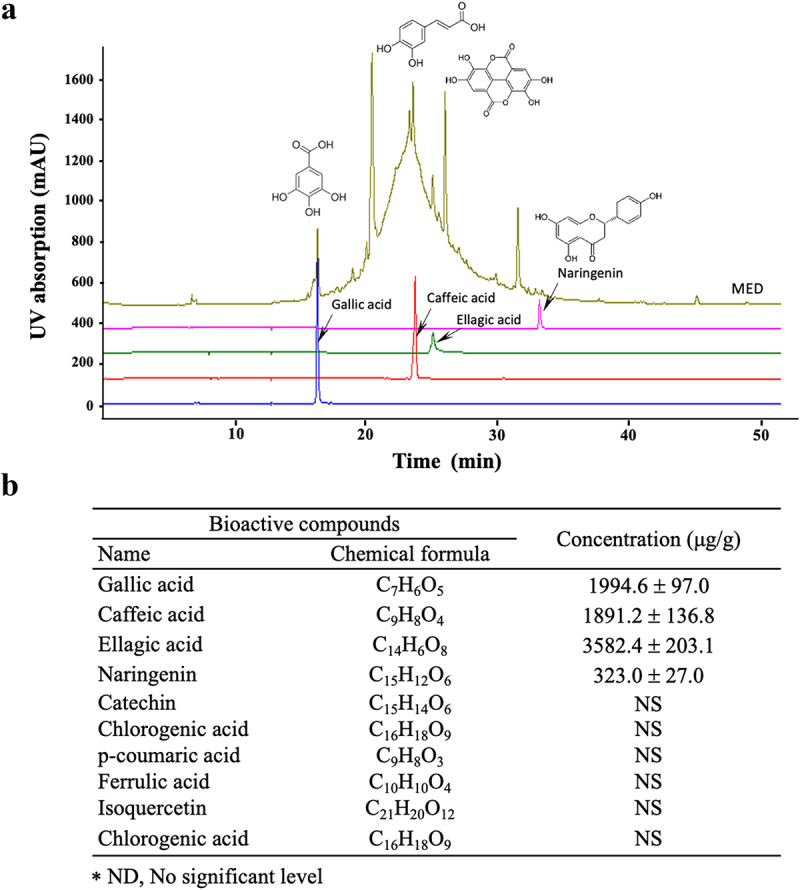
Phenolic compounds of MED. (a) LC-MS analysis of MED. Four components (gallic acid, caffeic acid, ellagic acid, and naringenin) were detected and shown as different peaks in the chromatogram. (b) Concentrations of the four phenolic compounds were calculated by comparing the peak of the standard substance with the peak of the MED sample. Three samples were assayed in duplicate by LC-MS analysis. Data are reported as the mean ± SD. Abbreviation: LC-MS/MS, liquid chromatography-tandem mass spectrometry.

### Histopathological analysis

Complex tissues containing bone, skin, and adjacent tissue were collected from the cranial defect zone of SD rats. These tissues were fixed overnight in 10% neutral buffered formaldehyde (pH 6.8), decalcificated with Gooding and Stewart fluid for a week [26], neutralized with 70% EtOH for a day, dehydrated using an alcohol series, and embedded in paraffin wax. The tissue sections (4 µm) were cut using a Leica microtome (Leica Microsystems), deparaffinized with xylene (DaeJung, Gyeonggi-do, Korea), rehydrated using graded ethanol (100 to 70%), washed with distilled water, stained with hematoxylin and eosin (H&E, Sigma-Aldrich Co.), and washed with dH_2_O. Histological alterations of cell adhesion and bone regeneration were observed in H&E-stained cranial defect sections using the Leica Application Suite (Leica Microsystems).

The distribution of mast cells in a tissue section was determined by performing toluidine blue staining as previously described [23]. Briefly, after deparaffinization and rehydration, skin sections on slides were stained with 0.25% toluidine blue (Sigma-Aldrich Co.) and then examined under an optical microscope to count mast cells. Numbers of mast cells per mm^2^ were determined using the Leica Application Suite (Leica Microsystems).

### Statistical significance analysis

Statistical analyses were performed using SPSS release 10.10 for Windows (IBM SPSS, SPSS Inc., Chicago, IL, USA). The significances of intergroup differences were determined by one-way analysis of variance followed by Tukey’s post hoc test for multiple comparisons. Results are presented as means ± SDs, and statistical significance was accepted for p values < 0.05.

## Results

### Active components in MED

**Figure 2.**
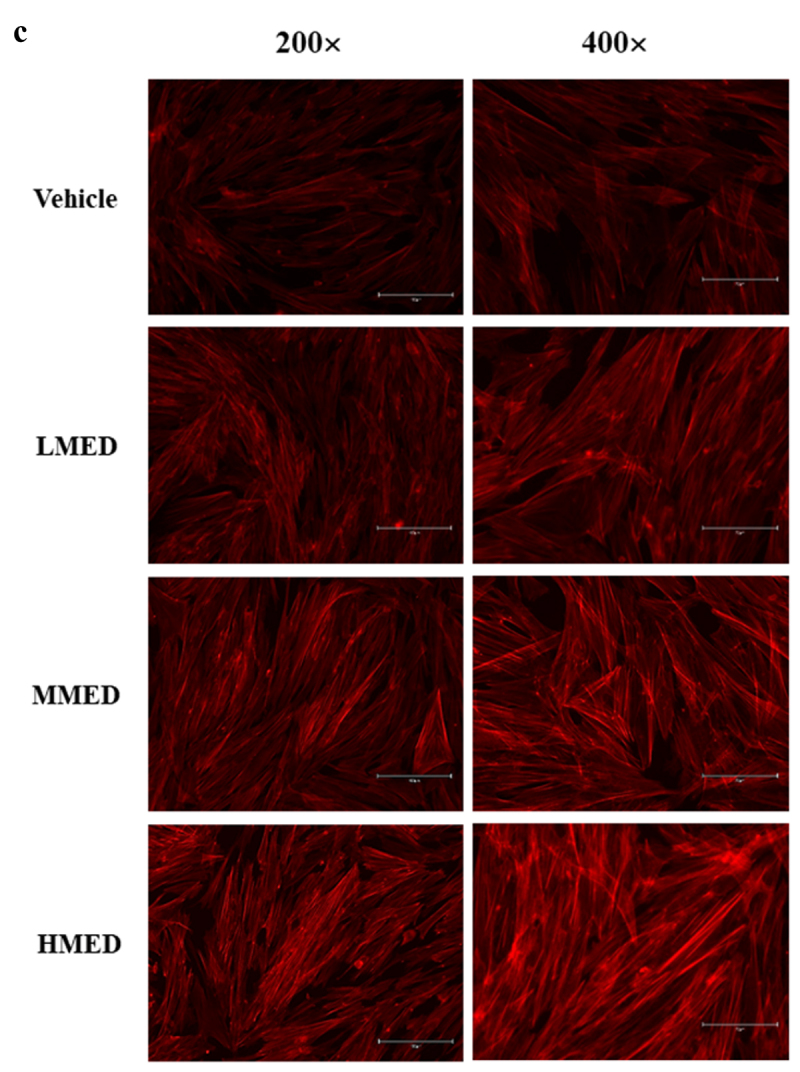
(Continued).

Firstly, we identified and quantified the active components in MED by examining the HPLC results. Among the 10 components analyzed, four (gallic acid, caffeic acid, ellagic acid, and naringenin) were detected at relatively high concentrations in MED. Ellagic acid was present at the highest concentration (3, 582.4 ± 203.1 μg/g), while gallic acid and caffeic acid concentrations were 1994.6 ± 97.0 and 1891.2 ± 136.8 μg/g, respectively. Of the four, naringenin was detected at the lowest level, 323.0 ± 136.8 μg/g (Figure 1(a-b)). However, no significant level was detected for the other six analyzed components (catechin, chlorogenic acid, p-coumaric acid, ferrulic acid, isoquercetin and chlorogenic acid) (Figure 1(b)). These results indicate that MED contains polyphenol and flavonoid components with strong anti-oxidative activities and has the potential for use as a novel stimulator of focal cell adhesion.
**Figure 2.**
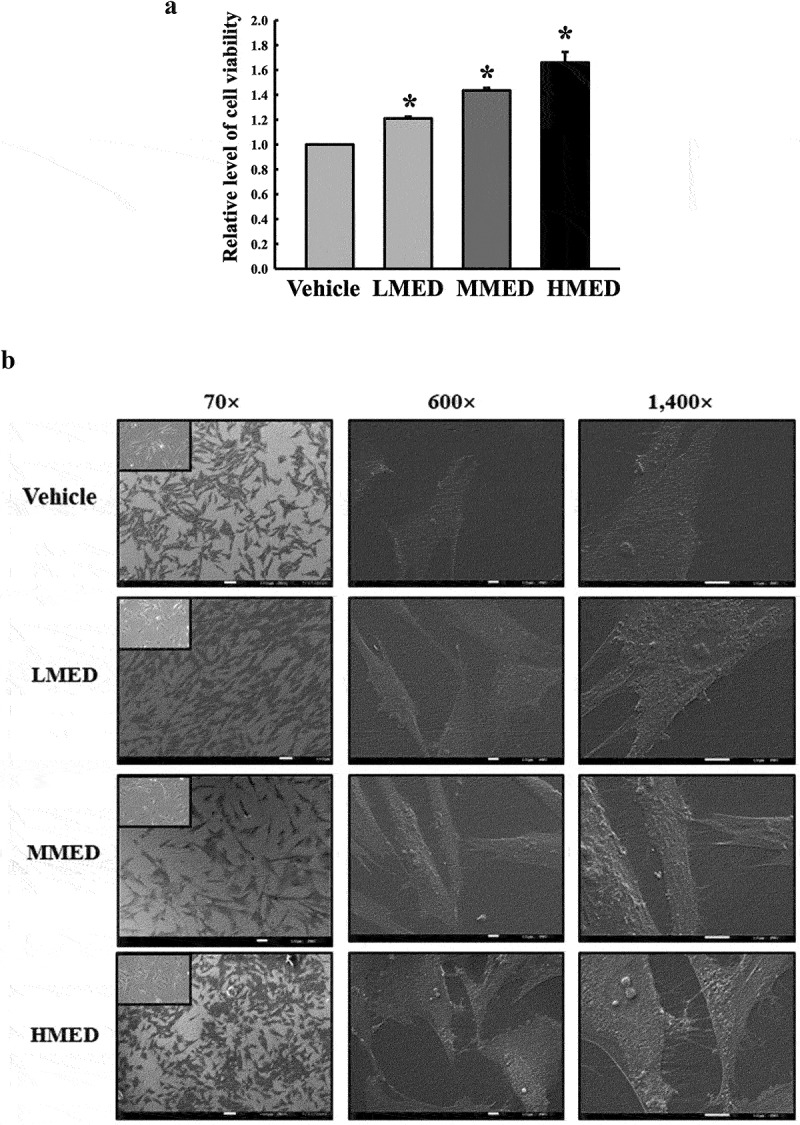
Cell variability, SEM and F-actin staining analyses of MED-treated NHDF cells. (a) After incubation of NHDF cells with 100, 200 or 400 μg/mL MED for 24 h, cell viability was analyzed as described in materials and methods. Three wells per group were used for the MTT assay, and optical density was measured in triplicate. Data are reported as the mean ± SD. *, p < 0.05 relative to the Vehicle-treated group. (b) The rectangle in the upper corner of the left column was morphological image of NHDF cells that observed under a microscope at 200× magnification. Also, SEM images were obtained from NHDF cells treated with 100, 200 or 400 μg/mL MED for 24 h under a SEM at 70×, 600×, and 1,400× magnification. (c) The cytoskeleton morphology of NHDF cells stained with ActinRed 555 Ready Probes were observed under a fluorescent microscope at 200x and 400x magnification. Abbreviations: SEM, Scanning electron microscope; MTT, 3-(4,5-Dimethylthiazol-2-yl)-2,5-Diphenyltetrazolium Bromide; LMED, Low concentration of MED; MMED, Middle concentration of MED; HMED, High concentration of MED.

### Effect of MED on proliferation activity and focal cell adhesion ability of NHDF cells

**Figure 3.**
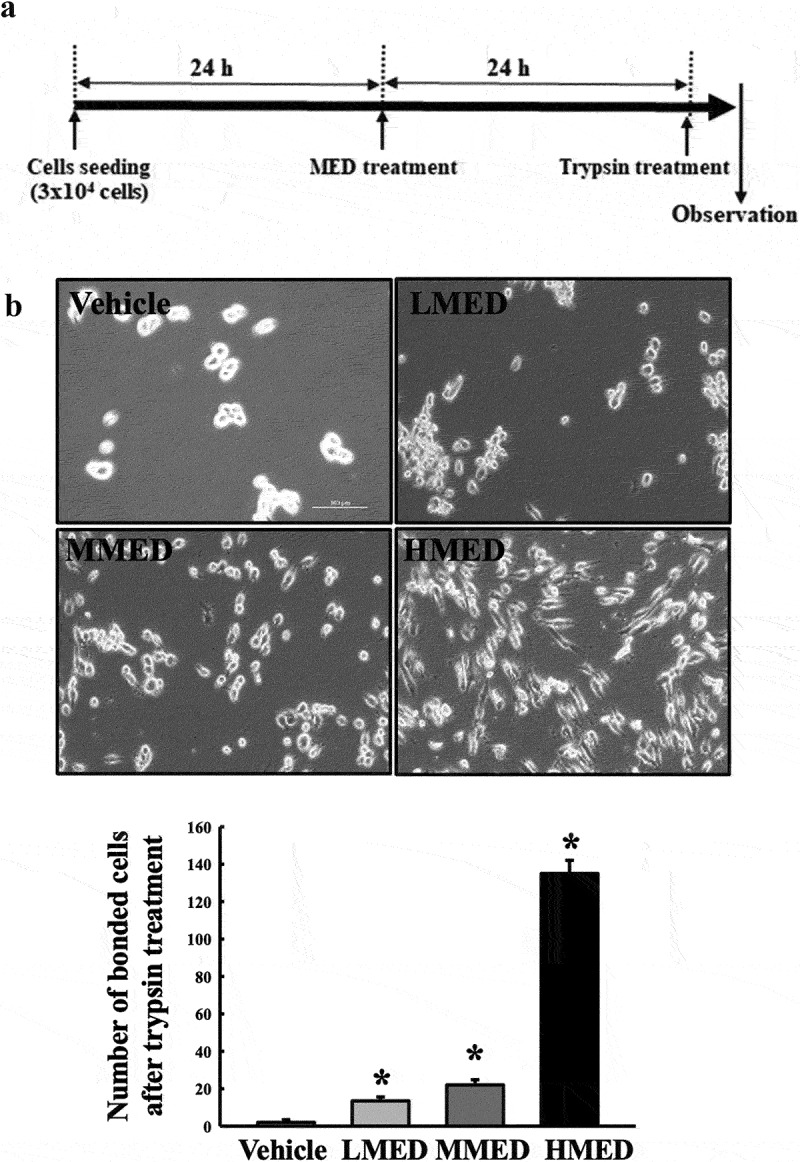
Trypsin-based cell detachment assay of MED-treated NHDF cells. (a) Schedule of trypsin-based cell detachment assay. (b) Image and number of trypsin-treated NHDF cells. Images and numbers of trypsin-treated NHDF cells were observed or counted after treatment of trypsin. Two to three wells per group were used for the detachment assay, and the number of cells was measured in duplicate. Data are reported as the mean ± SD. *, p < 0.05 relative to the Vehicle-treated group. Abbreviations: LMED, Low concentration of MED; MMED, Middle concentration of MED; HMED, High concentration of MED.

To investigate whether exposure to MED could affect the proliferation activity of cells, alterations in cell viability were examined in MED-treated NHDF cells. Cell viability significantly increased in a dose-dependent manner in the LMED-, MMED- and HMED-treated groups compared to the Vehicle-treated group (Figure 2(a)). Also, alterations on the morphological changes of cells and cytoskeletons were examined in SEM image and F-actin staining analysis of MED-treated NHDF cells in order to investigate whether exposure to MED could affect the focal cell adhesion ability of cells. In SEM image analysis, the number of dendrite-shaped structures and focal adhesion of cells were higher in the MED-treated group than in the Vehicle-treated group (Figure 2(b)). Also, F-actin cytoskeleton were abundantly distributed in the MED-treated group when compared to the Vehicle-treated group (Figure 2(c)). Furthermore, the focal cell adhesion ability was verified by trypsin detachment analysis. The number of attached cells was determined in MED-treated NHDF cells after trypsin treatment for 5 min to confirm the adhesion ability of MED. The number of attached cells was remarkably higher in the LMED-, MMED- and HMED-treated groups than in the Vehicle-treated group. The highest level was detected in the HMED-treated group (Figure 3). These results indicate that MED treatment may be associated with increases in proliferation activity and focal cell adhesion ability of NHDF cells.

**Figure 4.**
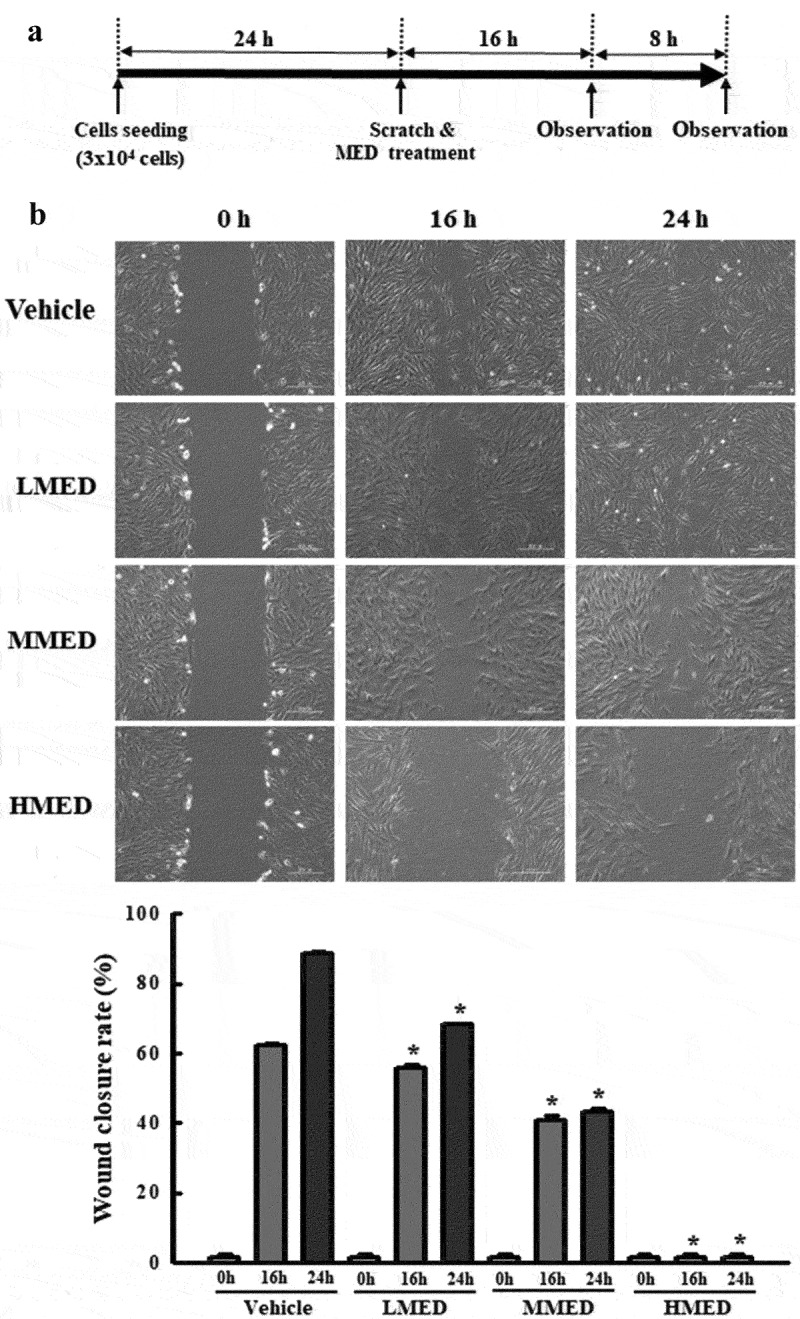
Analysis of cell migration in MED-treated NHDF cells. (a) Schedule of cell migration assay. (b) The migration ability of NHDF cells was analyzed by using the wound healing assay after treatment with 100, 200 or 400 μg/mL of MED. Morphological images (200× magnification) of cells were captured after 16 h and 24 h of incubation. Two to three wells per group were used for preparing the artificial injury, and closure rates were calculated in duplicate for each sample. Data are reported as the mean ± SD. *, p < 0.05 relative to the Vehicle-treated group. Abbreviations: LMED, Low concentration of MED; MMED, Middle concentration of MED; HMED, High concentration of MED.

### Suppressive effect of MED on migration activity of NHDF cells

We next examined whether the focal cell adhesion-stimulating effect of MED in NHDF cells is accompanied by suppression of the migration activity of these cells. To achieve this, the number of migrated cells in the NHDF scratch area was determined after treatment with MED. The wound closure rate significantly decreased in a dose-dependent manner after MED treatment (Figure 4). The results suggest that the cell adhesion-stimulating effects of MED may be closely linked to the suppression of migration activity in NHDF cells.

**Figure 5.**
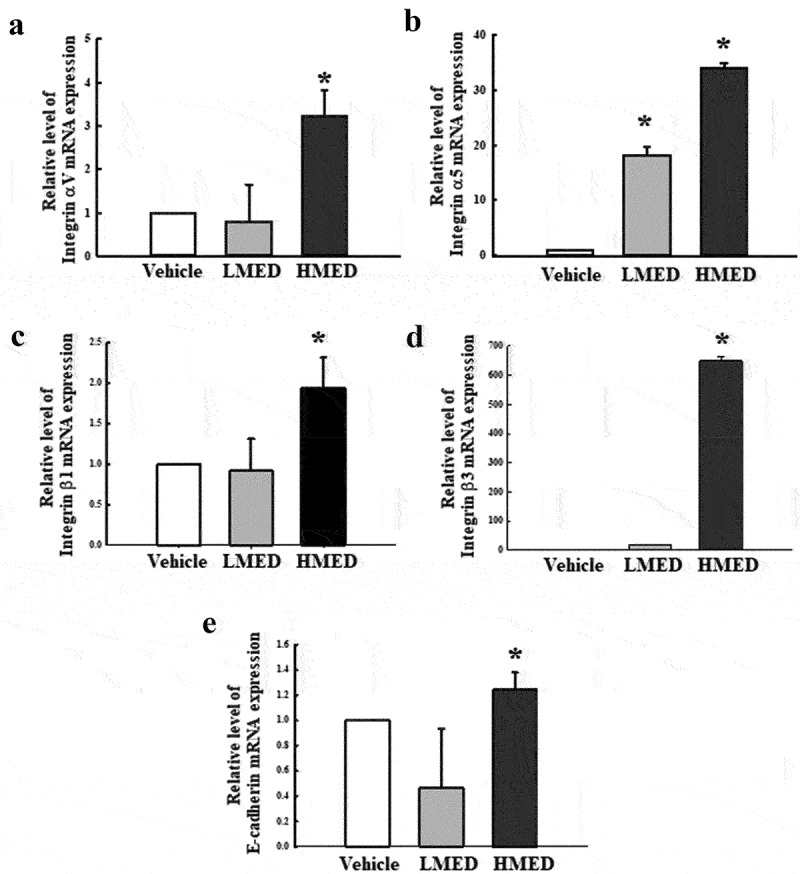
Expression of integrin and E-cadherin in MED-treated NHDF cells. The levels of integrin αV (a), integrin α5 (b), integrin β1 (c), integrin β3 (d), and E-cadherin (e) transcripts were detected in the total mRNA of NHDF cells by performing quantitative real time-PCR (qRT-PCR) with specific primers. Two to three dishes per group were used to prepare the total RNAs, and qRT-PCR was performed in duplicate for each sample. Data are reported as the mean ± SD. *, p < 0.05 relative to the Vehicle-treated group. Abbreviations: LMED, Low concentration of MED, HMED, High concentration of MED.

### Effect of MED on expression of adhesion receptor genes in NHDF cells

To investigate whether the focal cell adhesion-stimulating effect of MED in NHDF cells is accompanied by enhancements in the expression of adhesion receptor genes, alterations of the transcription levels of integrin family and E-cadherin genes were measured in MED-treated NHDF cells. The transcriptions of four integrin genes (integrin αV, integrin α5, integrin β1, and integrin β3) were significantly enhanced in the MED-treated groups, although the increases varied. Among the genes, integrin β3 had the highest expression level in the HMED-treated group compared to the Vehicle-treated group (Figure 5(a-d)). A similar but lower increase was observed in the expression of E-cadherin gene following HMED treatment, but the expression in the LMED-treated group remained unchanged. (Figure 5(e)). These results indicate that the focal cell adhesion-stimulating effect of MED may be associated with expression enhancement of integrin family and E-cadherin genes in NHDF cells.

**Figure 6.**
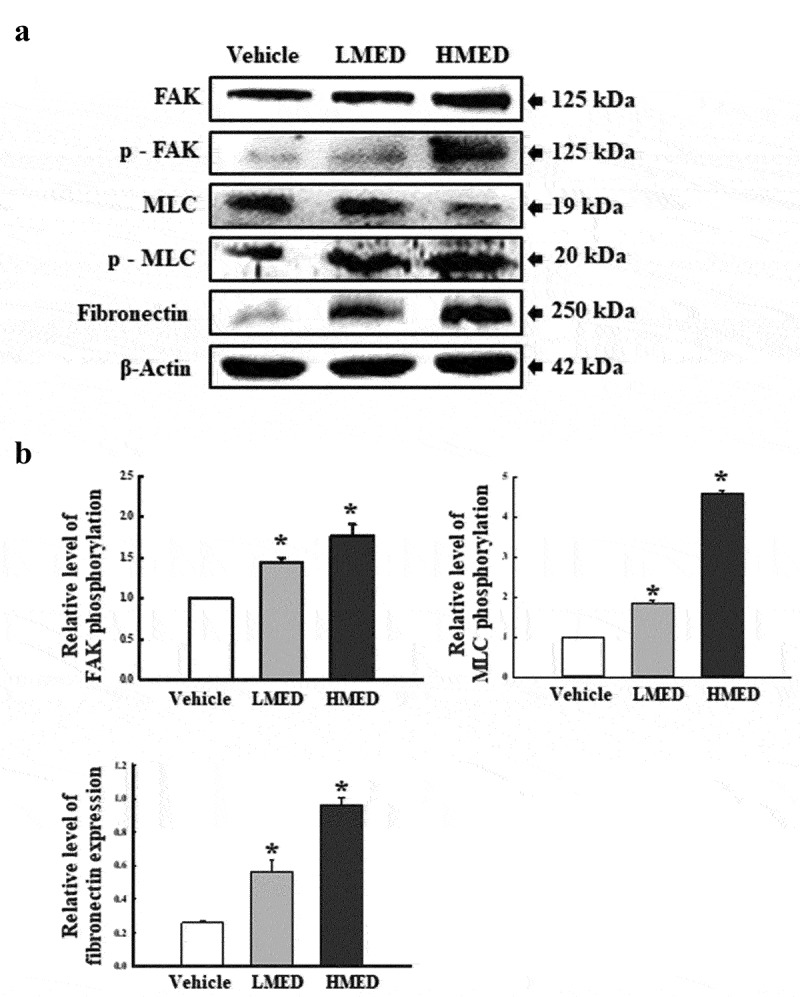
Expression of FAK/MLC2 signaling pathway members in MED-treated NHDF cells. Expression levels of five proteins were determined using an imaging densitometer. The level of each protein was presented relative to the intensity of actin. Two to three dishes per group were used to prepare cell homogenates, and western blot analysis was performed in duplicate for each sample. Data are reported as the mean ± SD. *, p < 0.05 relative to the Vehicle-treated group. Abbreviations: LMED, Low concentration of MED, HMED, High concentration of MED.

### Effect of MED on integrin downstream signaling pathway regulation in focal adhesion in NHDF cells

**Figure 7.**
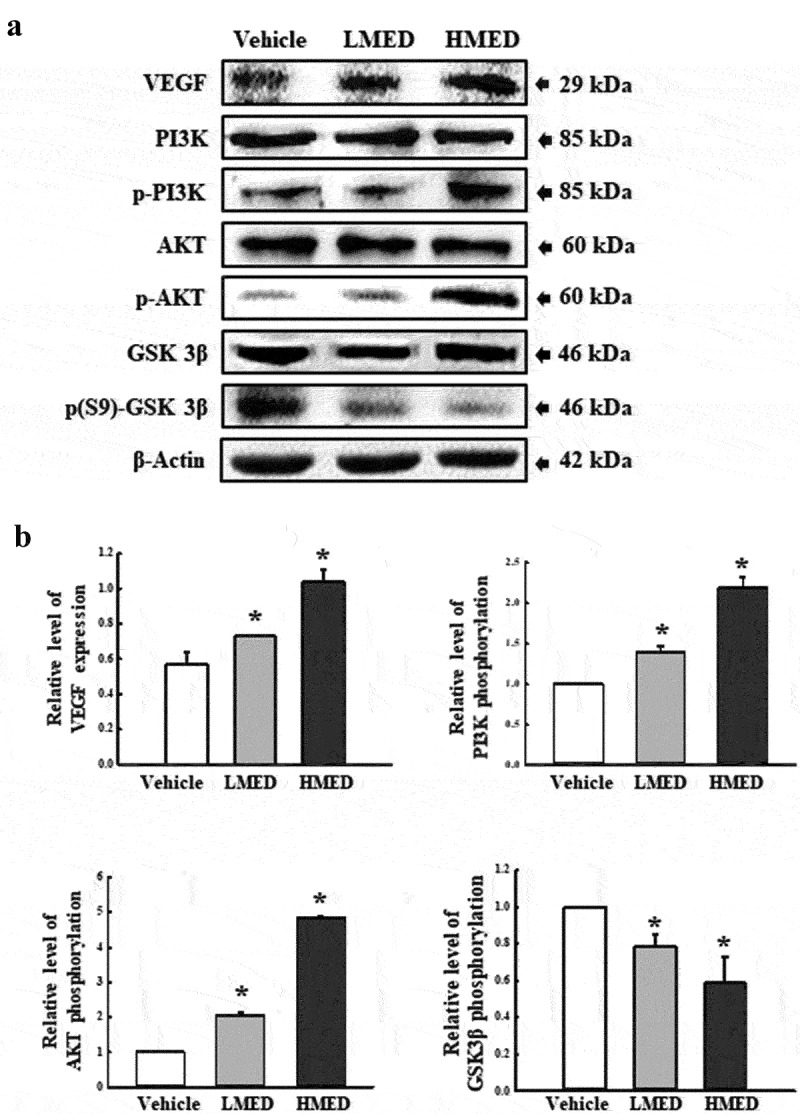
Expression of PI3K/Akt signaling pathway members in MED-treated NHDF cells. Expression levels of seven proteins were determined using an imaging densitometer. The level of each protein is presented relative to the intensity of actin. Two to three dishes per group were used to prepare cell homogenates, and western blot analysis was performed in duplicate for each sample. Data are reported as the mean ± SD. *, p < 0.05 relative to the Vehicle-treated group. Abbreviations: LMED, Low concentration of MED, HMED, High concentration of MED.

FAK has a key role in the modulation of protein recruitment and initiation of the MLC/Akt signaling pathway within the integrin downstream signaling pathway for focal cell adhesion [19]. To investigate whether the increased expressions of integrin genes induced by MED treatment are accompanied by regulation of the MLC2/FAK/Akt signaling pathway, alterations in the expressions of members of the FAK/MLC and PI3K/Akt signaling pathways were measured in NHDF cells after MED treatment. Phosphorylation levels of FAK and MLC in the FAK/MLC signaling pathway were increased by MED treatment in a dose-dependent manner; a similar increase was detected in fibronectin expression (Figure 6). Furthermore, the above alteration patterns were similarly observed in PI3K/Akt signaling pathway. In the MED-treated group, a dose-dependent increase was observed in VEGF expression, PI3K phosphorylation, and Akt phosphorylation, although GSK 3β phosphorylation was decreased (Figure 7). These results indicate that the stimulating effects of MED on integrin expression may be associated with activation of the MLC2/FAK/Akt signaling pathway in NHDF cells during the enhancement of focal cell adhesion.

**Figure 8.**
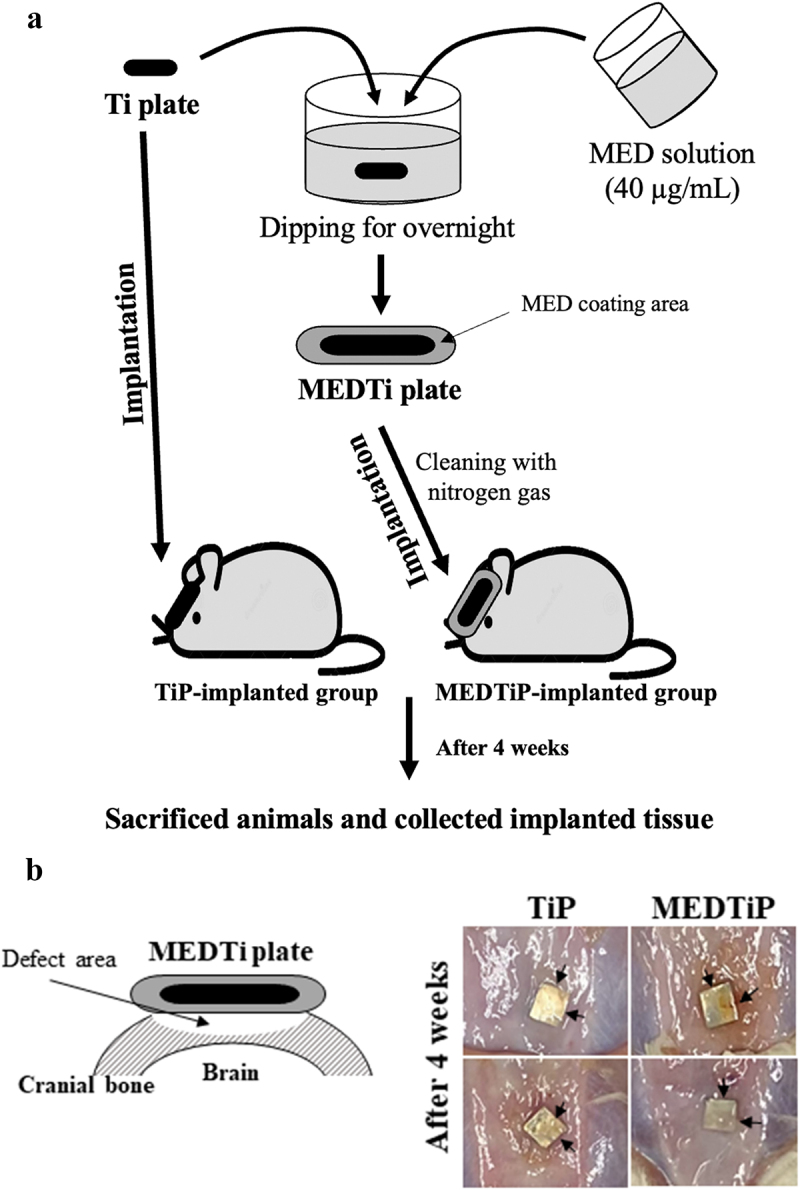
Experimental scheme for MEDTiP implantation. (a) After preparation of MEDTiP using a dipping method, TiP or MEDTiP were implanted in calvarial defects of SD rats for 4 weeks. (b) Image of the implanted region. After 4 weeks, TiP and MEDTiP were successfully attached to the defect region without any significant signs of injury. Abbreviations: TiP, Titanium plate, MEDTiP, MED-coated titanium plate.

### Stimulation effect of MED on focal cell adhesion in a calvarial defect rat model

**Figure 9.**
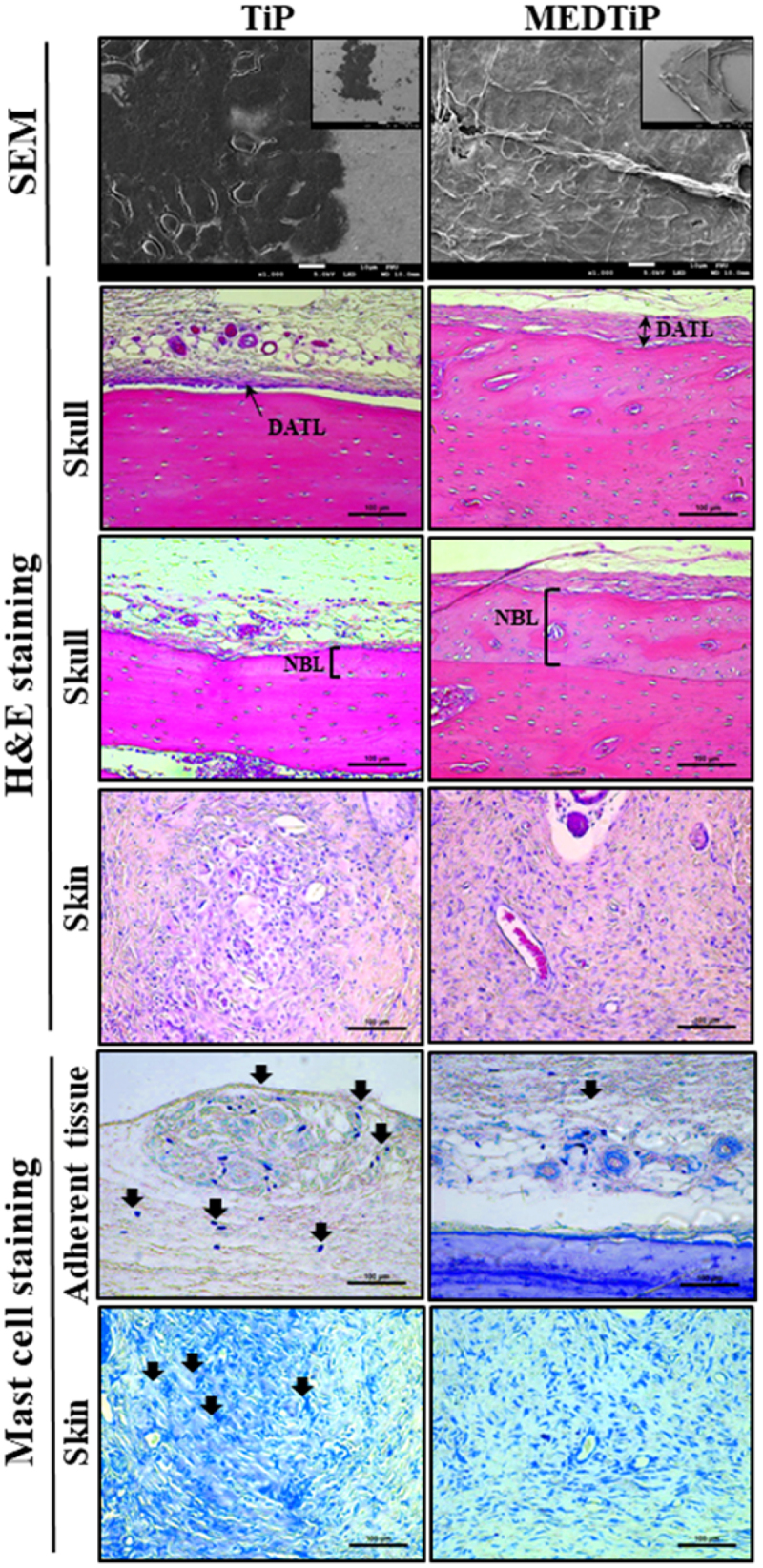
Histopathological structure of MEDTiP-implanted tissues. Adhesive changes were detected in sputter-coated MEDTiP at 100× (right corner) and 1,000× magnifications and in H&E-stained MEDTiP-implanted sections at 400× magnification. Infiltration of mast cells was identified in slide sections of skin and adherent tissue stained with toluidine blue, followed by observation at 400× magnification. Arrows indicate infiltrated mast cells in tissue adjacent to the MEDTiP implant. Three to four rats per group were used in the preparation of tissue slides, mast cell observing was performed in duplicate for each sample. Abbreviations: TiP, Titanium plate; MEDTiP, MED-coated titanium plate; DATL, Densely adhered tissue layer; NBL, New bone layer.

Next, we investigated whether the focal adhesion-stimulating effect of MED in NHDF cells could be reproduced in a calvarial defect rat model implanted with MEDTiP. To achieve this, MEDTiP was prepared by soaking the MED solution onto the surface of a Ti plate. The MED-treated plates were implanted into a calvarial defect in SD rats, and after 4 weeks, cell adhesion patterns were analyzed within the recipient site. As shown Figure 8(b), TiP and MEDTiP were successfully implanted to the defect site of the rat skull. In H&E-stained tissue samples, the densely adhered tissue layer was much thicker in the MEDTiP-implanted group than in the TiP-implanted group. Well-integrated fibrosis tissues were observed in the MEDTiP group. Furthermore, the formation of new bone and the number of mature osteocytes were significantly increased in the MEDTiP-implanted group compared to that in the TiP-implanted group. A significant decrease in mast cell infiltration was observed in the fibrous layer of the MEDTiP-implanted group (Figure 9). These results reveal that the MED released from a MEDTiP implant can enhance focal cell adhesion within the implantation site of the cranial defect model without significant inflammation.

### Effect of MED on the expression of adhesion receptor genes in a calvarial defect rat model

**Figure 10.**
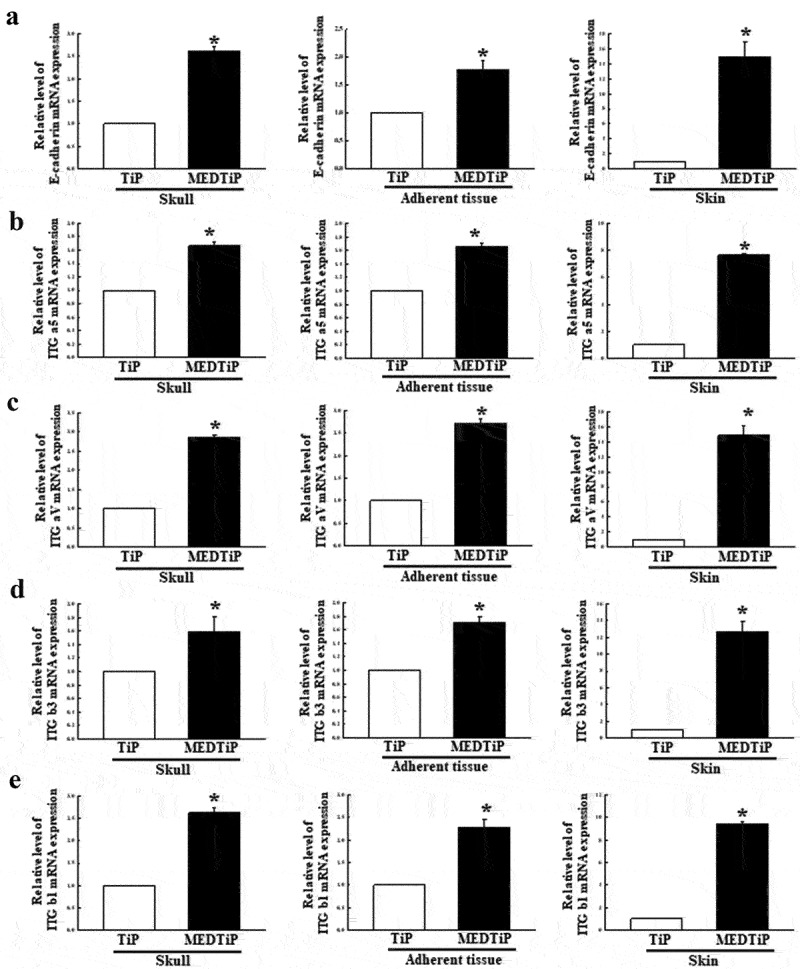
Expression of integrin and E-cadherin in MEDTiP-implanted calvarial defect rats. The levels of integrin αV (a), integrin α5 (b), integrin β1 (c), integrin β3 (d), and E-cadherin (e) transcripts were detected in the total mRNA of three different tissue types by performing quantitative real time-PCR (qRT-PCR) with specific primers. Two to three rats per group were used to prepare the total RNAs, and qRT-PCR was performed in duplicate for each sample. Data are reported as the mean ± SD. *, p < 0.05 relative to the TiP-implanted group. Abbreviations: TiP, Titanium plate, MEDTiP, MED-coated titanium plate, ITG, integrin.

To investigate whether the adhesion-stimulating effect of MED in the calvarial defect rat model was accompanied by enhancements in the expression of adhesion receptor genes, alterations in the transcription levels of integrin family and E-cadherin genes were measured in three different tissues (skull, adherent tissue, and skin) of MEDTiP-implanted rats. The transcription levels of four integrin genes (integrin αV, integrin α5, integrin β1, and integrin β3) were remarkably higher in the three different tissue types of the MEDTiP-implanted groups compared to the levels in the TiP-implanted group, although the increases varied by tissue type. Among the tissues examined, skin had the highest expression levels of all integrin genes (Figure 10). A similar increase pattern in the expression of the E-cadherin gene was observed in the MEDTiP-implanted groups (Figure 10). These results suggest that the adhesion-stimulating effect of MED may be associated with the enhancement of integrin family and E-cadherin gene expressions in the calvarial defect rat model after MEDTiP implantation.

### Effect of MED on integrin downstream signaling pathway regulation in focal adhesion in a calvarial defect rat model

**Figure 11.**
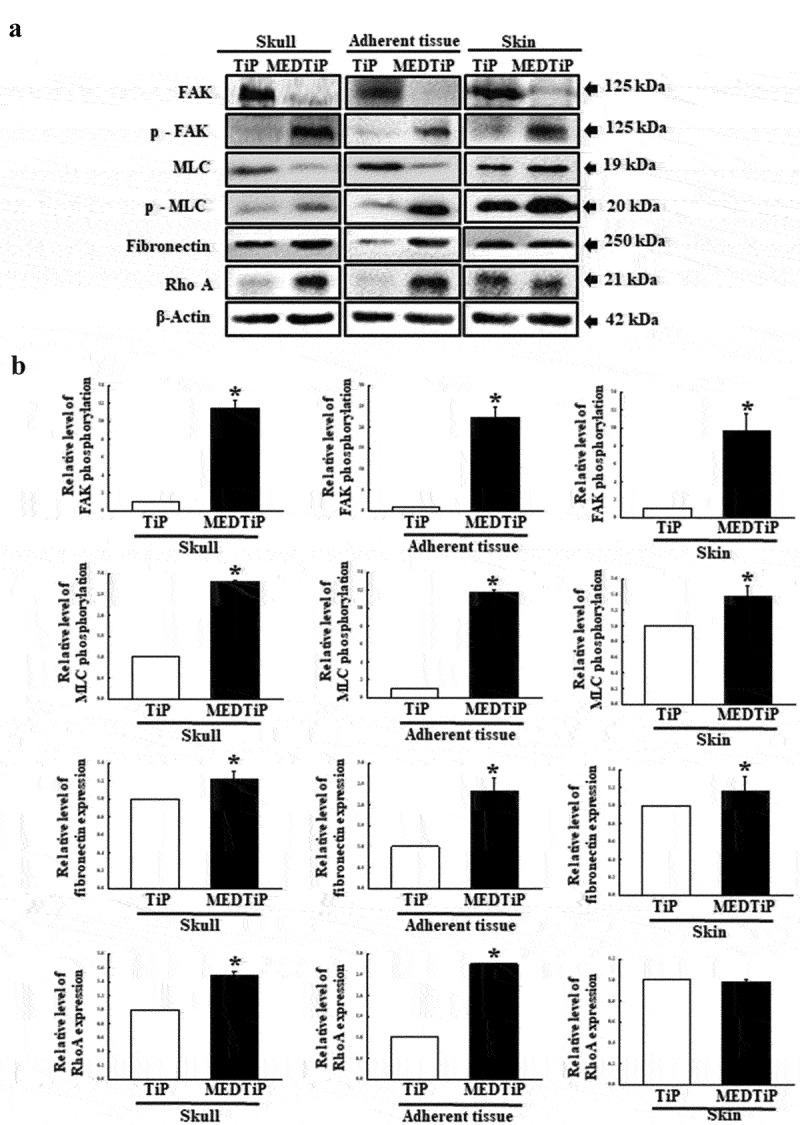
Expression of FAK/MLC2 signaling pathway members in MEDTiP-implanted calvarial defect rats. Expression levels of six proteins in three different tissue types were determined using an imaging densitometer. The level of each protein is presented relative to the intensity of actin. Two to three rats per group were used to prepare tissue homogenates, and western blot analysis was performed in duplicate for each sample. Data are reported as the mean ± SD. *, p < 0.05 relative to the TiP-implanted group. Abbreviations: TiP, Titanium plate, MEDTiP, MED-coated titanium plate.

**Figure 12.**
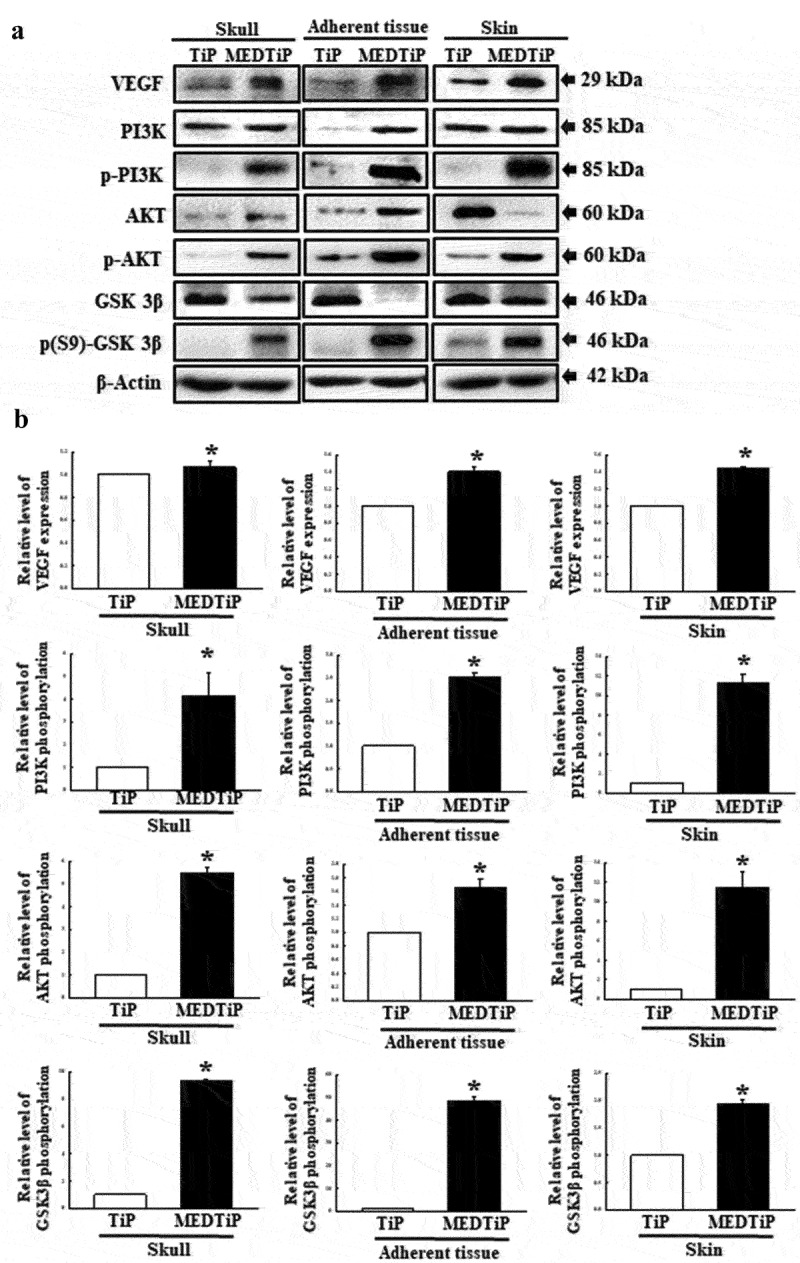
Expression of PI3K/Akt signaling pathway members in MEDTiP-implanted calvarial defect rats. Expression levels of seven proteins in three different tissue types were determined using an imaging densitometer. The level of each protein is presented relative to the intensity of actin. Two to three rats per group were used to prepare tissue homogenates, and western blot analysis was performed in duplicate for each sample. Data are reported as the mean ± SD. *, p < 0.05 relative to the TiP-implanted group. Abbreviations: TiP, Titanium plate, MEDTiP, MED-coated titanium plate.

Finally, we investigated whether the increased expressions of integrin genes induced by MEDTiP implantation are accompanied by alterations in the MLC2/FAK/Akt signaling pathway. Thus, alterations in the expressions of members of the FAK/MLC and PI3K/Akt signaling pathway were measured in three different tissues of the calvarial defect rat model after MEDTiP implantation. As shown Figure 11, phosphorylation levels of FAK and MLC in FAK/MLC signaling pathway were higher in the MEDTiP-implanted group than in the TiP implanted group, regardless of tissue type. A similar increase was detected in the fibronectin and RhoA expression levels in all three tissue types, except RhoA expression did not change in skin. Furthermore, changes in the regulation of members of the PI3K/Akt signaling pathway were very similar to those in the FAK/MLC signaling pathway. The phosphorylation levels of PI3K, Akt, and GSK 3β were remarkably higher in the MEDTiP-implanted group than in the TiP-implanted group. Notably, the highest increase in phosphorylation was observed in skin tissue. A similar increase in VEGF expression was observed in the MEDTiP-implanted group (Figure 12). These results indicate that the increased expression of integrin genes induced by MEDTiP implantation may be associated with activation of the MLC2/FAK/Akt signaling pathway in three different tissue types of the calvarial defect rat model after MEDTiP implantation.

## Discussion

Most studies on *D. tuberculatus* have been focused on its environmental ecology in forests and the composition of individual trees [27,28]. A few studies have analyzed the compositional components of different parts of *D. tuberculatus* and investigated the components’ anti-inflammatory functions in LPS-stimulated RAW cells and an EtOH-HCl-induced acute gastritis model [29,30]. However, little research has been conducted on the novel functions of *D. tuberculatus* and although it contains various components. To identify novel functions of this plant, we examined the stimulatory effects of *D. tuberculatus* on focal cell adhesion in NHDF cells and the calvarial defect rat model. Our results provide novel evidence that MED can enhance focal cell adhesion during MED treatment in NHDF cells and MEDTiP implantation in a calvarial defect rat model. Furthermore, the results of the present study show that MED can be considered a novel natural product that promotes focal cell adhesion, although more studies are needed to elucidate the effects of single components of MED.

*D. tuberculatus* may be known as *D. cordatus* or *D. grandifolius* in Cambodia, India, Laos, Thailand, and Vietnam [31]. On the other hand, the chemical constituents of this species have been unreported until now. However, the constituents of other species within the same family have been well described [32]. Phenolic acid derivatives, β-sitosterol, and bergenin were first identified in the stem and bark of *D. tuberculatus* [33]. In this study, we conducted HPLC analyses using MED to confirm the existence of several components. Four polyphenols (gallic acid, caffeic acid, ellagic acid, and naringenin) were present at significant levels in MED. These polyphenols, along with flavonoids and phenolic acid, are the most numerous and widely distributed group of bioactive molecules. Moreover, they have been shown to have various biological and pharmacological activities in many chronic diseases such as cancer, diabetes, Alzheimer’s disease, inflammation, and cardiovascular diseases [34]. Therefore, the results of the present study provide substantial evidence of novel functions of *D. tuberculatus*.

To that end, we examined the focal adhesion-stimulating effects of MED in NHDF cells and a calvarial defect rat model. MED treatment promoted the focal cell adhesion of NHDF cells by enhancing adhesion receptor expression and activation of an integrin downstream signaling pathway. These effects of MED were also observed in the calvarial defect rat model after implantation of a MEDTiP. Regulation on focal cell adhesion activity has been observed in various cell types after treatment with some natural products, although most of these effects are associated with cancer metastasis. Migration of VSMCs was remarkably inhibited after MWE treatment [20], while integrin-mediated focal adhesion was decreased in a B. chinense polysaccharide-treated group [21]. *Panax notoginseng* saponins and tea polyphenols have induced decreases in integrin family expression levels and inhibition of SMC-ECM interaction [22,24]. Cell migration was remarkably increased in human umbilical vein endothelial cells after treatment of *C. unshiu* peel [23]. Our results from MED-treated NHDF cells were very similar with those of *C. unshiu*-treated cells, although the major constituents and extractive solvent of the two products are different.

Integrin has a key structural role in connecting the ECM and the intracellular actin cytoskeleton at focal contacts [35]. Such interactions can initiate downstream signaling events for cell migration, growth, and survival [27]. The integrin derived cellular response is implicated in MLC2/FAK/Akt signaling in many types of cells [36,37,38]. The phosphorylation of MLC2 regulates the actin-myosin II interaction in non-muscle cells, while FAK mediates the integrin-regulated signal transduction associated with cell adhesion, migration, and proliferation [39,40]. Some studies have investigated the role of natural products in regulating the MLC2/FAK/Akt signaling pathway, although most of them have been focused on anti-cancer effects. Garcinol from *Garcinia indica* and *Ganfukang* induced decreases in the phosphorylation of FAK and Akt in colorectal cancer cells and hepatic fibrosis rat model respectively [41,42]. Also, similar effects were observed after treatments with a *Scutellaria baicalensis* and *Fritillaria cirrhosa* combination, as well as with *Gleditsia sinensis, Cortex Dictamni*, and Mulberry polyphenol [43,44,45,46]. However, contrasting results were reported in one study. The phosphorylation levels of FAK and ERK1/2 were significantly increased in human umbilical vein endothelial cells after treatment with *C. unshiu* peel [23]. The *C. unshiu* treatment results were very similar to those in the present study, although the cells investigated differed.

Our study provides additional scientific evidence of a novel natural product that can stimulate the MLC2/FAK/Akt signaling pathway. It is well known that several natural products have beneficial effects on various bone defects, including fractures and cracks, although there are differences in application methods for those products. Significant bone formation and regeneration were detected in a 1-wall intra-bone defect dog model treated with safflower seed extract and a parietal defect rabbit model treated with *Danshen* [47,48]. Porous bone substitutes incorporating *Danggui Buxue Tang* (DBT) stimulated the regenerative capacity of bone in a calvarial defect mice model without any significant complications, whereas the amount of bone formation and the number of osteoblast cells and osteoclasts were remarkably increased in a calvarial defect rat model after treatment with a *Nigella sativa* seed extract [25,49]. Similar beneficial effects on bone formation and regeneration were observed in an *Epimedium sagittatum* extract-treated calvarial defect rat model and a Dalbergia sissoo-treated drill-hole injury model [50,51]. The bone repair process and implant osseointegration were improved with *Chenopodium ambrosioides* extract and proanthocyanidin-rich grape seed extract treatments in animal models [52,53]. In support of the above research, our study results suggest that *D. tuberculatus* has beneficial effects on bone regeneration in a calvarial defect rat model. However, the animal treatment application methods differ between our research and previous research. In our study, a MEDTiP, MED-coated type, was used to evaluate MED effects on bone formation, whereas oral administration of natural products or implantation of simple mixtures containing polymers and natural products were administered in previous studies.

## Conclusion

This study examined alterations in proliferation activity, cell adhesion ability, adhesion receptor expression, and the integrin downstream signaling pathway both *in vitro* and *in vivo*. The results of the present study provide evidence that MED can successfully stimulate focal cell adhesion, integrins and E-cadherin expression, and the FAK/MLC2 and PI3K/Akt signaling pathways in NHDF cells and in a calvarial defect rat model. The stimulatory effects of MED on focal cell adhesion of NHDF cells and bone defect rats indicate the potential of MED as a stimulator for biomaterials-cell interactions. However, more studies are required to elucidate the effects of single components of MED and the molecular mechanisms responsible for these effects.

## Data Availability

Data openly available in a public repository that issues datasets with DOIs.
